# Enhanced presentation of MHC class Ia, Ib and class II-restricted peptides encapsulated in biodegradable nanoparticles: a promising strategy for tumor immunotherapy

**DOI:** 10.1186/1479-5876-9-34

**Published:** 2011-03-31

**Authors:** Wenxue Ma, Trevor Smith, Vladimir Bogin, Yu Zhang, Cengiz Ozkan, Mihri Ozkan, Melanie Hayden, Stephanie Schroter, Ewa Carrier, Davorka Messmer, Vipin Kumar, Boris Minev

**Affiliations:** 1 Moores UCSD Cancer Center, University of California San Diego; 2 Laboratory of Autoimmunity, Torrey Pines Institute for Molecular Studies; 3 MediStem, Inc., San Diego, CA; 4 Laboratory of Biomaterials and Nanotechnology, University of California Riverside; 5 Division of Neurosurgery, University of California San Diego; 6 Genelux Corporation, San Diego, CA

## Abstract

**Background:**

Many peptide-based cancer vaccines have been tested in clinical trials with a limited success, mostly due to difficulties associated with peptide stability and delivery, resulting in inefficient antigen presentation. Therefore, the development of suitable and efficient vaccine carrier systems remains a major challenge.

**Methods:**

To address this issue, we have engineered polylactic-co-glycolic acid (PLGA) nanoparticles incorporating: (i) two MHC class I-restricted clinically-relevant peptides, (ii) a MHC class II-binding peptide, and (iii) a non-classical MHC class I-binding peptide. We formulated the nanoparticles utilizing a double emulsion-solvent evaporation technique and characterized their surface morphology, size, zeta potential and peptide content. We also loaded human and murine dendritic cells (DC) with the peptide-containing nanoparticles and determined their ability to present the encapsulated peptide antigens and to induce tumor-specific cytotoxic T lymphocytes (CTL) *in vitro*.

**Results:**

We confirmed that the nanoparticles are not toxic to either mouse or human dendritic cells, and do not have any effect on the DC maturation. We also demonstrated a significantly enhanced presentation of the encapsulated peptides upon internalization of the nanoparticles by DC, and confirmed that the improved peptide presentation is actually associated with more efficient generation of peptide-specific CTL and T helper cell responses.

**Conclusion:**

Encapsulating antigens in PLGA nanoparticles offers unique advantages such as higher efficiency of antigen loading, prolonged presentation of the antigens, prevention of peptide degradation, specific targeting of antigens to antigen presenting cells, improved shelf life of the antigens, and easy scale up for pharmaceutical production. Therefore, these findings are highly significant to the development of synthetic vaccines, and the induction of CTL for adoptive immunotherapy.

## Background

In recent years, peptides derived from tumor-associated antigens (TAA) have been identified for a variety of human cancers [1]. Thus far, however, effective peptide vaccination of patients with cancer has been limited to very few trials [2]. The relative paucity of responsiveness after conventional peptide vaccination is mostly due to the high levels of protein degradation, limiting antigen delivery. Polymeric nanoparticles (NP) may allow encapsulation of peptides inside a polymeric matrix, protecting them against enzymatic and hydrolytic degradation. In addition, the nanoparticle vaccine approach offers the possibility of developing tailor-made vaccines containing specific targets or molecules that may improve their function [3].

Dendritic cells (DC) are the most potent professional antigen-presenting cells (APC), having the ability to initiate primary immune responses [4]. Therefore, immunotherapy utilizing DC has become a promising therapeutic modality in recent years [5-7]. However, the lack of efficient and long-lasting antigen presentation by DC *in vivo *has been a major difficulty in the development of effective vaccines. These obstacles could be circumvented through the development of nanoparticles, which can efficiently deliver the antigenic peptides into the APC.

Our recent studies have characterized distinct subsets of regulatory CD4+FOXP3- and CD8αα+TCRαβ+ T cells that target autoaggressive Vβ8.2+ T cell responses for down-regulation and protection against autoimmune disease [8-13]. Several non-classical MHC class I, Qa-1a-restricted CD8αα+TCRαβ+ T cell and MHC class II-restricted CD4+ T cell clones and lines have been characterized [8,9,11]. The CD4+ T cells recognize a TCR-derived peptide in the context of a class II MHC molecule, I-Au [11]. However, CD8αα+TCRαβ+ T cells are cytotoxic and recognize another TCR-derived peptide bound to a class Ib molecule, Qa-1 [8,9]. These cells are physiologically primed and operate in unison to assist in recovery from T cell-mediated experimental autoimmune encephalomyelitis in H-2^u ^mice [10,13,14]. Interestingly, recent data suggest that class Ib MHC-restricted cytotoxic CD8+ T cells also play an important role in anti-tumor immunity [15,16]. Therefore, it is important to examine whether CTL can be effectively primed using nanoparticle containing class Ib-binding peptides.

To engineer our nanoparticle-based vaccines, we utilized the biodegradable and biocompatible polymer PLGA, which is already approved by the US FDA [17,18]. PLGA is easy to formulate into different devices and has generated immense interest because of its favorable properties, which include good biocompatibility, biodegradability, and mechanical strength [17,18].

Here we demonstrate for the first time efficient nanoparticle-facilitated loading of class I-restricted, clinically relevant TAA-derived peptides to human DC, and the development of nanoparticles incorporating MHC class II and non-classical MHC class I, Qa-1-binding peptides that are able to stimulate helper CD4+ and cytotoxic CD8αα+TCRαβ+ T cells. Importantly, we confirmed that the enhanced peptide presentation by the NP-loaded DC is associated with more efficient generation of antitumor CTL.

## Methods

### Antibodies and reagents

Antibodies: Anti-human IFN-γ (mAb 1-D1K) - Mabtech Inc. (Mariemont, OH), anti-HLA-A2-FITC, anti-HLA-DR-PE, anti-CD83-PE, anti-CD80-FITC, anti-CD86-FITC, mouse-IgG1-FITC, mouse-IgG1-PE, and mouse-IgG2a-PE all from BD (San Diego, CA). Cytokines: GM-CSF - BERLEX (Richmond, CA), Interleukin 4 (IL-4) - PeproTech (Rocky Hill, NJ), IL-2 - Chiron (Emeryville, CA). Peptides: MART-1_27-35_, gp100_209-217 _and mSTEAP_326-335 _- GenScript Corp. (Piscataway, NJ), murine TCR Vβ8.2 chain peptides: B5 (76-101) - Caltech (Pasadena, CA), and p42 (42-50) - Synthetic Biomolecules (San Diego, CA). PLGA - Birmingham Polymers (Birmingham, AL), bovine serum albumin (BSA) and Poly (vinyl alcohol) (PVA) - Sigma-Aldrich (St. Louis, MO), coumarin 6 - Polyscience (Warrington, PA).

### Cell lines

T2 cell line [19] was purchased from ATCC (Manassas, VA). Human melanoma cell lines 624 and 1351, as well as human tumor-infiltrating lymphocytes (TIL) cell lines TIL1235 and TIL1520 were kindly provided by Dr. John R Wunderlich (NIH/NCI, Bethesda, MD). CD4+TCRαβ+ (B5.1) and CD8αα+TCRαβ+ (XT14), [8] T cell lines were generated in H-2u background and were specific for the Vβ8.2TCR-derived peptides, B5 and p42-50, respectively. The CD4+TCRαβ+ (B5.1) cell line was generated from the draining lymph nodes of a H-2u mouse immunized i.p. with 20 μg of TCR peptide B5.

### Nanoparticle formulation

Peptide-containing NP were formulated using a double emulsion-solvent evaporation technique as we described previously [20]. For optimizing the peptide dose entrapped in the NP, 300 μg, 600 μg and 1 mg of each peptide was formulated into the PLGA polymer in each NP batch. Some NP were formulated with a fluorescent dye (coumarin 6) by adding 100 μg of coumarin 6 to the polymer solution prior to emulsification.

### Nanoparticle characterization

To characterize the surface morphology of the NP we utilized a scanning electron microscope (SEM). Particle size analysis and zeta potential determination was carried out using a Zetasizer (Malvern, Worcestershire, UK). The peptide content of the peptide-loaded NP was determined by HPLC using a C18 column (Waters, Milford, MA). Specifically, the peptides and nanoparticles were separated and identified using ultraviolet (UV) detection and known standards, at a wavelength of 280nm (attenuation 0.002 AU). An aliquot (50 μl) was injected onto the column and eluted with a mobile phase containing a gradient mixture of reagent A, 0.1% trifluoroacetic acid (TFA) in water (Sigma Aldrich St Louis, MO, USA), and reagent B, 0.1% TFA in Acetonitrile. The gradient times were as follows: 0-23 minutes, 75% A and 25% B; 23 -25 minutes, 0% A and 100% B. Total run time was 25 minutes at a flow rate of 0.8 ml per minute.

### Generation of Human DC

Peripheral blood mononuclear cells (PBMC) isolated from buffy coats of healthy donors were allowed to adhere in 6-well plates for 1 hour. The adherent cells were cultured with 1000 U/ml GM-CSF and 300 U/ml IL-4, with cytokines added on days 2, 4, and 6. Lipopolysaccharide (LPS) was added to the culture medium on day 7, and two days later, the mature DC (mDC) were harvested and characterized by FACS using antibodies against HLA-DR, CD80, CD83, and CD86.

### Generation of murine bone marrow-derived dendritic cells (BMDC)

Murine DC were derived from tibias and femurs by flushing out the bone marrow with RPMI medium as described [21] and cultured with 10 ng/ml IL-4 and 25 ng/ml GM-CSF for 5 days, with cytokines added on day 3.

### Nanoparticle uptake imaging studies

Human immature DC (imDC) were seeded overnight on sterile cover slips and incubated with NP containing coumarin-6 for 1-hr at 37°C. Then, the cover slips were washed and observed with a fluorescent microscope. For confocal microscopy, imDC were incubated in 4-well chamber slides for 1 hour at 37°C with 100 μg/ml Coumarin 6-containing NP, washed and fixed with paraformaldehyde. After washing and staining with DAPI, imDC were mounted on glass slides. Confocal images were obtained with a Leica TCS SP2 UV confocal microscope.

### FACS analysis of NP-loaded DC

NP-loaded and non-loaded DC were stained with the following antibodies for 30 min at 4°C: PE-anti-human HLA-DR, PE-anti-human CD83, FITC-anti-human CD80, and FITC-anti-human CD86. All data was analyzed using the Cell Quest software.

### Antigen presentation by the NP-loaded DC

Human imDC were collected and pulsed with the peptides MART-1_27-35_, or gp100_209-217_, or incubated for 1 hour with 100 μg/ml nanoparticles formulated with the same peptides at 300 μg, 600 μg or 1 mg peptide per batch. LPS (100 ng/ml) was subsequently added. Two days later, the mDC were harvested, tested by FACS, and co-cultured for 20 hours with TIL1235 (recognizing MART-1_27-35_) or TIL1520 (recognizing gp100_209-217_), and the efficiency of the antigen presentation was evaluated in an IFN-γ ELISPOT assay.

### Induction of CTL with the NP-loaded mDC

The mDC were mixed with HLA-A2^+^/CD8^+ ^T cells at a ratio of 1:10 in complete medium, and incubated at 37°C. Four days later, 20 U/ml of IL-2 and 30 U/ml of IL-7 were added. On days 7 and 14, the cultures were re-stimulated with peptide-pulsed adherent autologous CD8^− ^cells in complete medium. Specifically, irradiated CD8^− ^cells were incubated for 2 hours with β-2 microglobulin (at 5 μg/ml) and peptide (at 5 μg/ml), washed once and used as stimulators of the CTL. Seven days later, the CTL were tested by IFN-γ ELISPOT assays and CytoTox96 cytotoxicity assays.

## Elispot

The frequency of cytokine-secreting cells was measured in a human IFN-γ ELISPOT assay as we previously described [22]. The responder cells (TIL1235, TIL1520 or CTL) were incubated with the DC cultures at a ratio of 1:1 for 20 hours, and the spots were counted using ImmunoSpot® (CTL, Cleveland OH).

### Cytotoxicity Assays

The CTL cytotoxic activity was determined by CytoTox96 cytotoxicity assays (Promega) according to manufacturer's protocol.

### Nanoparticle stimulation assays using murine BMDC

BMDC were harvested after 5 days culture in IL-4 and GM-CSF, and incubated with 100 μg/ml nanoparticles (B5 or control) or 10 μg/ml B5 peptide for one hour. DC were then removed from nanoparticle/peptide supernatant by positive selection using anti-CD11c beads (Miltenyi), and split into aliquots. Day 0 DC aliquots were used straight away in a cell proliferation assay and 2x10^4 ^B5-reactive CD4+ T cells (B5.1) were co-cultured with 3.3 to 100x10^3 ^DC. A 72-hour assay, with ^3^H-thymidine added for the last 8 hours was performed. Day 2 DC aliquots were cultured for 2 days in GM-CSF and IL-4, and a proliferation assay was performed. For the p42-50 nanoparticle assay imDC were incubated with 100 and 200 μg/ml of p42-50 NP or control NP, or with 20 μg/ml p42-50 peptide. DC were then removed from nanoparticle/peptide supernatant by positive selection using anti-CD11c beads, treated with LPS for 12 hours, washed, and co-cultured in proliferation assays with 2 × 10^4 ^CD8+ (XT14) T cells [8]. At 48 hrs, supernatants from the assay wells were removed and IFN-γ measured by ELISA.

### Statistical analysis

Data were analyzed by descriptive statistics, calculating the mean and standard deviation for continuous variables. The paired Student's *t *test was used to evaluate differences between NP-loaded versus non-loaded pairs of cell cultures. The *P *values of <0.05 were considered significant.

## ResultS

### Nanoparticle characterization

**Figure 1:**
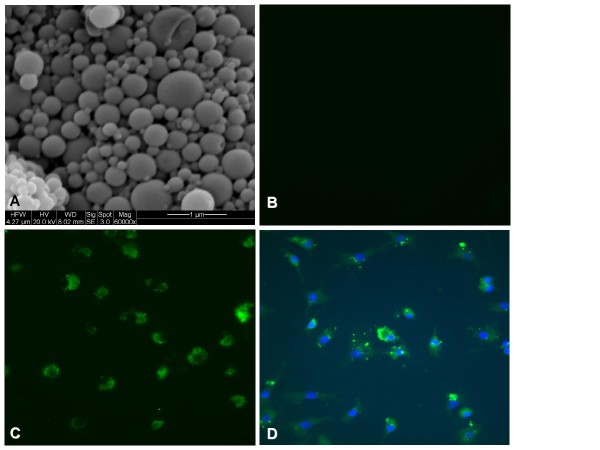
**Nanoparticle internalization by immature DC**. **(A)** Nanoparticles observed with a SEM. Magnification 60,000×. NP-loaded imDC examined under a fluorescence microscope after a 1-h incubation with free coumarin-6 **(B)**, or with NP containing coumarin-6 **(C)**. NP-loaded imDC incubated with Hoechst nuclear stain **(D)**. Magnification 400×.

SEM images revealed that the nanoparticles were spherical in shape, with a smooth surface (Figure 1A). The size distribution of the nanoparticles was in the range of 181-282 nm, with a mean diameter of 215.46 ± 48.6 nm, and an average zeta potential of -20.2 mV.

The peptide content of three different nanoparticle preparations, as determined by HPLC, is (i) 1.588 micrograms of peptide per milligram of the nanoparticle preparation formulated with 300 micrograms of peptide, (ii) 3.176 micrograms of peptide per milligram of the nanoparticle preparation formulated with 600 micrograms of peptide, and (iii) 5.293 micrograms of peptide per milligram of the nanoparticle preparation formulated with 1000 micrograms of peptide. In preliminary experiments we selected NP formulation prepared with 600 μg of peptide for our *in vitro *immunization experiments. As we routinely use 100 μg/ml peptide-loaded NP for DC-loading, this amount corresponds to 0.3176 μg of peptide per ml of DC-loading medium. In comparison, the amount of peptide used for pulsing of the control DC group is 1 μg/ml or about three times as much as those in the NP formulation.

### Human dendritic cells can efficiently internalize nanoparticles

**Figure 2:**
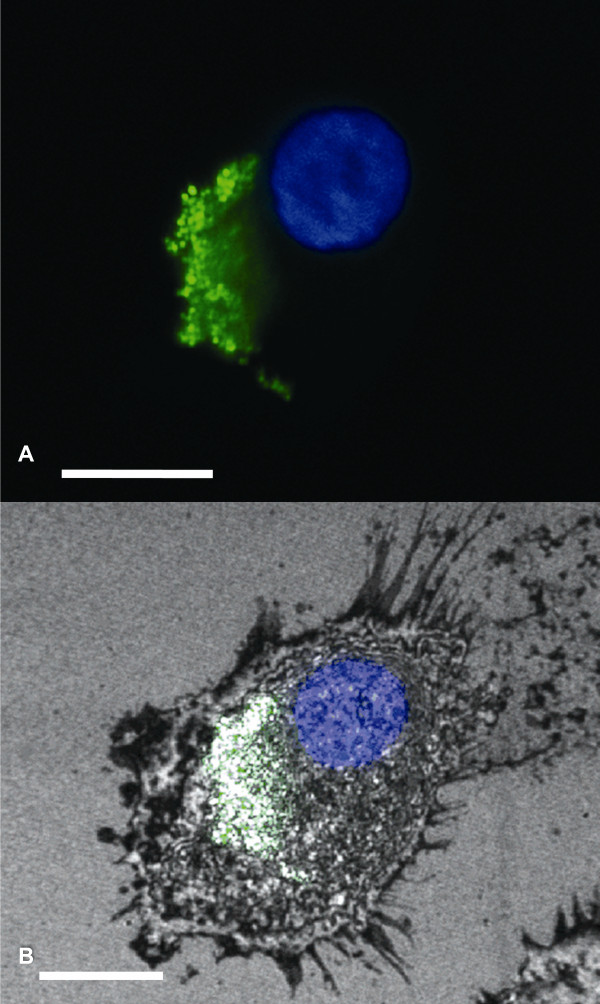
**Confocal microscope analysis**. A single immature DC observed after a 1-hour incubation with NP containing peptide Mart-1_27-35 _and coumarin-6. Overlaid confocal images using DAPI, FITC **(A)**, and reflection **(B) **channels are shown. The bar represents 10 μm.

Coumarin-6-containing NP were visible inside DC after just 1-hour of co-incubation (Figure 1C). However, no fluorescence was observed when the same DC were incubated with free coumarin-6 (Figure 1B). These studies showed that 100% of the observed human imDC internalized nanoparticles. Nuclear staining revealed that the NP were most likely localized in the cytoplasm or endoplasmic reticulum of the DC (Figure 1D). Confocal microscopy using the coumarin-6-containing NP revealed an intense cytoplasmic fluorescence (6-coumarin) in the DC (Figure 2), confirming that our peptide-containing NP are avidly internalized by the imDC.

### Effect of PLGA nanoparticle uptake on the maturation status of the human DC

**Figure 3:**
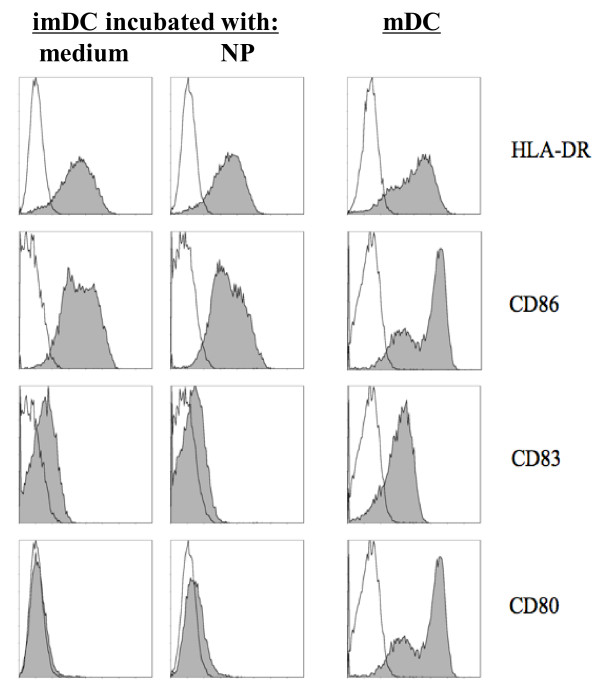
**Phenotype of NP-loaded DC**. Immature DC (imDC) analyzed 30 hours after a 1-hour incubation with NP containing Mart-1_27-35 _and coumarin-6, and compared to mature LPS-stimulated DC (mDC). Open area plots - DC stained with isotype controls; solid area plots - DC stained with antibodies for HLA-DR, CD80, CD83, and CD86.

The tested DC surface markers CD80, CD83, CD86 and HLA-DR were not upregulated after incubation with our PLGA nanoparticles in several repeated experiments (Figure 3). In contrast, incubation with LPS induced significant upregulation of these markers. These results indicate that the NP uptake did not influence DC phenotype and their ability to mature.

### Enhanced antigen presentation by human DC loaded with NP containing class I-restricted peptides

**Figure 4:**
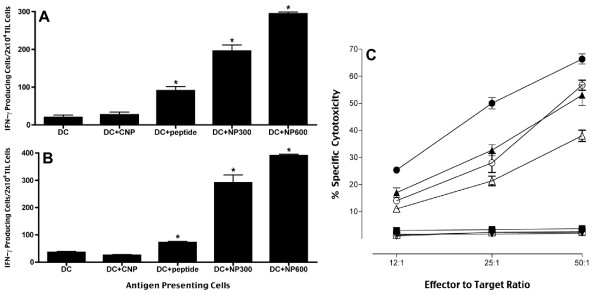
**Enhanced antigen presentation and CTL induction by NP-loaded DC**. DC loaded with nanoparticles containing the peptides: **(A) **MART-1_27-35_, or **(B) **gp100_209-217_. DC were incubated with: soluble peptide (DC+peptide); empty nanoparticles (CNP); or with nanoparticles formulated with the same peptides using 300 μg (DC+NP300) or 600 μg (DC+NP600) peptide per batch. Four days later, DC were co-cultured for 20 hours with TIL1235 (recognizing MART-1_27-35_) or TIL1520 (recognizing gp100_209-217_) cells, and the antigen presentation was evaluated in an IFN-γ ELISPOT assay. **(C) **Cytotoxic activity of CTL induced *in vitro *with peptide-pulsed or NP-loaded dendritic cells: Dendritic cells were pulsed with the peptide MART-1_27-35 _or with MART-1_27-35_-containing NP and used as APC to induce MART-1_27-35_-specific CTL. The experimental groups include: T2 target cells incubated with peptide-DC induced CTL (□), or with NP-DC induced CTL (■); peptide-pulsed T2 cells incubated with peptide-DC induced CTL (∓), or with NP-DC induced CTL (ℓ); HLA-A2^+ ^melanoma cells 624 incubated with peptide-DC induced CTL (△), or with NP-DC induced CTL (▲); and HLA-A2^- ^melanoma cells 1351 incubated with peptide-DC induced CTL (▽), or with NP-DC induced CTL (▼). The CTL lines were incubated with the target cells for 4 hours and the cytotoxicity was determined with a standard LDH-release assay (Promega). Data is representative of 3 independent experiments; *bars*, SD. *, significant differences (*P *< 0.05) between experimental and control cultures (non-pulsed DC or CNP-loaded DC).

We prepared NP containing the peptides MART-1_27-35_, and gp100_209-217_. Peptide-pulsed or NP-loaded DC were compared for their ability to present these peptides to TIL lines recognizing MART-1_27-35_, (TIL1235) and gp100_209-217 _(TIL1520) four days after the NP-loading. NP-loaded DC were recognized by TILs much better than the DC pulsed with the same peptides or DC loaded with empty nanoparticles (Figure 4A, B). These findings suggest that the significantly enhanced presentation of the peptides loaded with NP resulted from improved loading and sustained release of these peptides after the internalization of the NP.

### Improved generation of peptide-specific CTL with NP-loaded DC

To address the question whether enhanced presentation of the peptides actually improves the generation of CTL, we initiated *in vitro *cultures of NP-loaded DC with responder CD8+ T lymphocytes. Specifically, we determined whether our NP vaccines formulated with the clinically-relevant melanoma peptide MART-1_27-35 _could induce potent CTL capable of recognizing and killing peptide-pulsed target cells and melanoma tumor cells *in vitro*. CTL induced with peptide-pulsed DC were compared to CTL induced with NP-loaded DC (Figure 4C). We found that the CTL induced with the melanoma peptide MART-1_27-35 _encapsulated into our nanoparticles were able to recognize and kill specifically not only the peptide-pulsed T2 cells, but also the HLA-A2-positive melanoma cells 624. In contrast, the CTL induced with the peptide-pulsed DC were less efficient in killing these target cells, and the HLA-A2-negative melanoma cells 1351 were not recognized (Figure 4C). These experiments confirmed that our nanoparticle-based vaccine could expand precursor CTL in PBMC of HLA-A2+ donors and induce MHC class I-restricted, specific CTL responses against the melanoma cells.

### Enhanced presentation of murine MHC class II-restricted peptide encapsulated into nanoparticles

**Figure 5:**
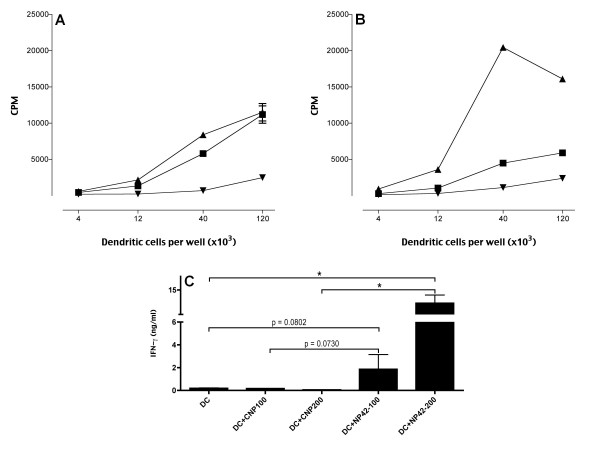
**Enhanced stimulation of MHC class II-restricted and non-classical MHC class I-restricted T cell lines by NP-loaded DC**. **(A-B) **MHC class II-restricted CD4+ T cell lines: imDC incubated with: 100 μg/ml NP containing B5 peptide (▲); empty nanoparticles (▼); or 10 μg/ml B5 peptide (■) for one hour. **(A) **B5-reactive CD4+ T cells co-cultured with imDC. A 72-hour assay was performed, with ^3^H-thymidine added for the last 8 hours. **(B) **DC cultured for another 48 hours before co-culture with the responder T cells and a ^3^H-thymidine assay. **(C) **MHC class I-restricted CD8+ T cell lines: imDC incubated with 100/200 μg/ml empty nanoparticles (CNP), or with nanoparticles containing the peptide p42 (NP42) for one hour. Subsequently, p42-reactive CD8+ T cells were co-cultured with DC, and 48-hour later, IFN-gamma was measured by ELISA. Data is representative of 3 independent experiments; *bars*, SD. *, Significant differences (*P *< 0.05) between experimental and control cultures.

Murine imDC were incubated for 1hr with B5-loaded nanoparticles (B5-NP), control nanoparticles or B5 peptide. Equivalent levels of proliferation in B5-reactive CD4+ T cell line, B5.1 on co-culture with B5-NP or peptide pulsed DC was observed (Figure 5A.) We had previously predicted that B5 peptide encapsulated into our NP would be protected from degradation and released slowly into the dendritic cell's antigen processing pathways. This would allow for an increased duration of antigen presentation compared to naked peptide that would be quickly degraded. To investigate this imDC were incubated with NP and peptide for 1 hr, before separation from the non-captured NP or peptide. DC were then incubated alone for 48 hrs before being co-cultured with the CD4+ T cell line, B5.1. Indeed, we observed an enhanced proliferation of the CD4+ T cells co-cultured with B5 nanoparticle-treated DC in comparison to the B5 peptide-treated DC (Figure 5B). This finding suggests that the nanoparticles increase the duration for which antigenic peptides can be presented by the DC.

### Presentation of murine nonclassical MHC class I, Qa-1-restricted peptide encapsulated into nanoparticles

imDC loaded with peptide p42 encapsulated into NP were co-cultured with Qa-1-resticted CD8αα+TCRαβ+T cell line (XT-14). We found that DC loaded with NP containing the peptide p42 could stimulate XT-14 T cell line to produce IFN-γ (Figure 5C). No significant stimulation was observed with the empty control NP. These experiments clearly show that imDC loaded with NP vehicles carrying non-classical MHC class I peptides can present efficiently these Qa-1-restricted peptides.

## Discussion

Epitope-based peptide vaccines can be designed to include multiple epitopes from one or several antigens, and can be easily analyzed for purity and produced economically on a large scale. Currently however, there are no human peptide-based cancer vaccines on the market, mostly resulting from difficulties associated with their poor immunogenicity, stability and delivery. We [22,23] and others [24,25] have described strategies to enhance the peptides' immunogenicity and stability. Direct peptide delivery to dendritic cells using particulate delivery systems is a promising new approach. In addition to having a depot effect on the peptide antigens, inherent properties of the particles themselves engender immunogenicity of the peptides, and allow uptake of an immunogenic package of peptides and other molecules [26]. This approach is exemplified with the use of liposomes and immunostimulatory complexes [27], as well as virosomes [28] and exosomes [29].

Cultured DC are very suitable for the delivery of peptide vaccines, as their direct loading bypasses the processing requirements and allows for precise delivery of peptide antigens to the immune system [30]. However, the potency of DC-based vaccines is significantly reduced by the short persistence of the MHC/peptide/β2-microglobulin complexes on the DC surface, especially when the antigen-derived peptides are bound from the outside and not processed [31].

It is therefore essential that the vaccine-carrier systems are capable of delivering the vaccines inside the APCs, in order to facilitate a potent and prolonged antigen presentation. Liposome-based systems are commonly used, but their delivery efficiency is sub-optimal, and the duration of their effects is relatively short [32]. In addition, the liposome is not a thermodynamically stable system and therefore has multiple physical stability issues such as rapid drug leakage, merging of vesicles and low loading efficiency. The viral vector delivery is limited by a difficult large-scale production and potential for toxicity [33], immune and inflammatory responses [34], as well as insertional mutagenesis and oncogenic effects [35].

Nanoparticle-based vaccine delivery systems offer significant advantages due to their safety profile, ease of manufacture and storage, and most importantly, their versatility in designing customized products for specific targeting applications [36]. We developed previously PLGA nanoparticles designed for sustained release of drugs or DNA to human umbilical vein endothelial cells [37], and prostate cancer cells [20]. In the current study, we used PLGA-based nanoparticles as a delivery system to load clinically-relevant, tumor antigen-derived peptides into human DC, and found that 100% of imDC internalized NP after just 1-hour co-incubation with the nanoparticles (Figure 2). Using our new PLGA nanoparticles containing MHC class I peptides as a vaccine delivery system offers distinct advantages over the administration of the corresponding soluble peptides. These NP are composed of solid PLGA polymers, therefore avoiding the drug-leakage problems involved in liposome formulations, thus preventing the proteolytic degradation of the antigen. These polymeric NP can also be easily lyophilized for long-term storage with better stability than liposome and other liquid carrier systems.

We also considered the influence of PLGA uptake on the properties of the DC, and its potential negative effect on some important parameters for the DC function. The hydrolysis of PLGA leads to the liberation of lactic and glycolic acids, and therefore we expected that the resulting acidification could negatively affect the cellular functions of the PLGA-loaded DC. It this study we did not observe any negative effects and/or reduced viability of the PLGA-loaded DC. We also found that our NP formulations did not have an effect on the maturation of human or murine DC. Our findings are in agreement with a similar study, which reported that immature DC loaded with PLGA particles exhibited a similar DC phenotype to those without any loading [38]. In contrast, another study showed that a maturation process has been induced by polystyrene nanospheres, as the maturation markers HLA-DR and CD86 were upregulated [39]. A similar result was observed using PLGA nanoparticle formulations in cord blood derived DC [40], as well as murine bone marrow derived DC [41]. These discrepancies are most likely due to the different culture conditions and differences in the nanoparticle preparations in these studies. We conclude that the intake of our PLGA NP does not adversely affect important DC functions required for their use as vaccines in the clinic.

In the present study, the efficiency of the antigen presentation by human DC was significantly enhanced after just 1-hour incubation with our NP containing class I-restricted peptides (Figure 4A and 4b). The level of antigen presentation was related to the amount of peptide incorporated inside the NP. Importantly, in these studies we used patient-derived TIL lines and peptides used in many clinical trials, which suggests a speedy utilization of these data in clinical trial designs.

For the presentation of MHC class I restricted T cell epitopes from PLGA-encapsulated peptides, the involved antigen presenting cells must be able to "cross present" the exogenous peptides onto MHC class I molecules by either the classical proteasome and TAP-dependent pathway [42], or by an alternative TAP-independent pathway [22,23] of antigen presentation. DC, macrophages and some endothelial cells have been shown to be able to cross present [43]. Cross presentation of soluble proteins by DC can occur, but it is extremely inefficient, as it usually requires the incubation with high concentrations of protein antigens. Remarkably, in this study the amount of peptides encapsulated inside our NP was significantly lower than the amount of peptides used to pulse the DC externally. Still, we observed a greatly enhanced antigen presentation by the NP-loaded DC. We suggest that this enhancement of antigen presentation is due to the slow hydrolysis of the PLGA NP in the endosomes the DC, which provides a continuous supply of peptide ligands for newly synthesized MHC class I and II molecules. We also demonstrated that the NP-loaded DC were able to induce more potent CTL than the DC pulsed with the same peptides externally (Figure 4C). The resulting CTL were able to recognize and kill efficiently not only peptide-pulsed target cells, but also HLA-matched melanoma cells expressing the corresponding antigens. Similarly, murine DC could present antigens more efficiently as a result of the antigen encapsulation inside our nanoparticles (Figure 5). These results provide further confirmation of the usefulness of this approach for induction of potent and specific anti-tumor responses, and its potential for clinical application.

Our nanoparticles were also effective in stimulating class II-restricted CD4+ T cells as well as non-classical class I-restricted CD8αα+TCRαβ+ cytotoxic T cells. Notably, these CD4+ and CD8αα+TCRαβ+ T cells are involved in the negative feed back regulation of autoimmunity [8,9,11-13]. Our earlier studies have shown that priming of these regulatory T cells following peptide or DNA immunization results in immune regulation [8,11,12,44]. Data presented in this paper further indicate that nanoparticles containing appropriate peptides can be used to generate effective vaccines not only against tumors but also in the intervention of autoimmune diseases. Class Ib MHC-restricted cytotoxic T cells also play an important part in the anti-tumor responses [15,16]. It is clear from our data that nanoparticles containing class Ib MHC (Qa-1 or HLA-E in humans) binding tumor-associated antigens can also be designed.

## Conclusions

The development of nanoparticle-based vaccines derived from clinically relevant tumor antigens holds great promise. Encapsulating antigens in PLGA nanoparticles offers unique advantages such as higher efficiency of antigen loading, prolonged presentation of the antigens, prevention of peptide degradation, specific targeting of antigens to APC, improved shelf life of the antigens, and easy scale up for pharmaceutical production. In addition, a variety of targeting strategies may be readily utilized, including ligand-receptor mediated targeting, antibody-antigen interaction, lectin-carbohydrate interaction, etc. Recent advances in polymer chemistry also allow for many variations of the nanoparticle design, including simultaneous delivery of a combination of vaccines, immunomodulators, drugs or other compounds, creating a potent multivalent therapeutic strategy. This paper is therefore highly significant to the development of optimized clinical grade vaccines, and the induction of CTL for adoptive immunotherapy of cancer.

## Abbreviations

APC: antigen-presenting cells; CTL: cytotoxic T lymphocytes; DC: dendritic cells; GM-CSF: granulocyte-macrophage colony-stimulating factor; HLA: human leukocyte antigen; MART-1: melanoma antigen recognized by T cell 1; NP: polymeric nanoparticles; PLGA: polylactic-co-glycolic acid; TAA: tumor-associated antigens; TAP: transporter associated with antigen processing; TCR: T cell receptor

## Competing interests

The authors declare that they have no competing interests.

## Authors' contributions

WM carried out and participated in all of the studies, including nanoparticle preparation and characterization, DC isolation and loading, CTL induction in vitro, data analysis and manuscript preparation. TS carried out murine DC isolation and loading, analysis of presentation of murine MHC class II-restricted peptides and murine non-classical MHC class I, Qa-1-restricted peptides encapsulated into nanoparticles, and data analysis. VB participated in the design of the study, helped with the statistical analysis and manuscript preparation. YZ participated in nanoparticle characterization, DC loading and imaging and data analysis. CO YZ participated in nanoparticle characterization, DC imaging and data analysis. MO participated in nanoparticle characterization, DC loading and imaging and data analysis. MH participated in DC loading and CTL induction. SS participated in DC loading and CTL induction. EC participated in the design of the study, helped with the statistical analysis and manuscript preparation. DM participated in nanoparticle characterization, DC loading and data analysis. VK participated in data analysis and supervised studies related to murine class II and Qa-1-restricted T cell presentation. BM designed, supervised and coordinated the study, performed the statistical analysis and drafted the manuscript. All authors read and approved the final manuscript.
